# Effects of hypoxic preconditioning on neuroblastoma tumour oxygenation and metabolic signature in a chick embryo model

**DOI:** 10.1042/BSR20180185

**Published:** 2018-08-29

**Authors:** Yousef K. Al-Mutawa, Anne Herrmann, Catriona Corbishley, Paul D. Losty, Marie Phelan, Violaine Sée

**Affiliations:** 1 Department of Biochemistry, Institute of Integrated Biology, University of Liverpool, Liverpool L69 7ZB, U.K.; 2 Academic Paediatric Surgery, Division of Child Health, University of Liverpool, Liverpool L12 2AP, U.K.; 3 Department of Molecular and Cellular Physiology, Institute of Translational Medicine, University of Liverpool, Liverpool L69 3GE, U.K.

**Keywords:** cell cultures, chick embryo, hypoxia, NMR spectroscopy, oxygenation, tumour biology

## Abstract

Hypoxia episodes and areas in tumours have been associated with metastatic dissemination and poor prognosis. Given the link between tumour tissue oxygen levels and cellular metabolic activity, we hypothesised that the metabolic profile between metastatic and non-metastatic tumours would reveal potential new biomarkers and signalling cues. We have used a previously established chick embryo model for neuroblastoma growth and metastasis, where the metastatic phenotype can be controlled by neuroblastoma cell hypoxic preconditioning (3 days at 1% O_2_). We measured, with fibre-optic oxygen sensors, the effects of the hypoxic preconditioning on the tumour oxygenation, within tumours formed by SK-N-AS cells on the chorioallantoic membrane (CAM) of chick embryos. We found that the difference between the metastatic and non-metastatic intratumoural oxygen levels was small (0.35% O_2_), with a mean below 1.5% O_2_ for most tumours. The metabolomic profiling, using NMR spectroscopy, of neuroblastoma cells cultured in normoxia or hypoxia for 3 days, and of the tumours formed by these cells showed that the effects of hypoxia *in vitro* did not compare with *in vivo* tumours. One notable difference was the high levels of the glycolytic end-products triggered by hypoxia *in vitro*, but not by hypoxia preconditioning in tumours, likely due to the very high basal levels of these metabolites in tumours compared with cells. In conclusion, we have identified high levels of ketones (3-hydroxybutyrate), lactate and phosphocholine in hypoxic preconditioned tumours, all known to fuel tumour growth, and we herein point to the poor relevance of *in vitro* metabolomic experiments for cancer research.

## Introduction

Neuroblastoma is the most common extracranial solid tumour in childhood and exhibits a broad spectrum of severity ranging from asymptomatic spontaneous regression to severe systemic illness, metastasis and death. Metastatic tumour development and disease relapse are strongly associated with poor patient survival in neuroblastoma and critically need to be addressed in terms of improved detection and therapeutic intervention, through biological and clinical investigations [1,2].

The occurrence of oxygen deprivation (hypoxia) in solid tumours is associated with cancer progression, treatment resistance and poor clinical outcome. Tumour hypoxia arises from a multifactorial aetiology ultimately resulting in O_2_ levels lower than the 2–9% physiological range found in most human tissues [3,4]. While O_2_ levels <2% are regarded as hypoxic in many normal tissues, tumour environments are more oxygen deprived with O_2_ levels detected at less than 0.7% O_2_ [5,6]. The neuroblastoma hypoxic signature has previously been correlated to patient outcome [7] and we have further demonstrated, utilising the chick embryo chorioallantoic membrane (CAM) model, that hypoxic preconditioning drives metastasis and the regulation of genes supportive of tumour dissemination [8]. In addition, we observed that hypoxic preconditioned cells are able to trigger the invasion of adjacent non-metastatic cells [8]. While tumour hypoxia has been extensively reported as a poor prognostic marker in disease, much remains to be discovered with regards to the intracellular modifications associated with hypoxia and their link with the metastatic phenotype [9,10].

Various indirect and direct methods have been used to characterise tumour oxygen levels. While indirect *ex vivo* methods often rely on injecting hypoxia markers and detecting them via flow cytometry or immunohistochemistry, direct *in vivo* methods have the advantage of providing spatial and temporal oxygen measurements. Such direct methods are more challenging, and often require invasive devices such as oxygen-sensitive microelectrodes or fibre-optic devices. Tumour oxygenation can also be assessed non-invasively using imaging techniques such as ^19^F MRI, pulsed EPR imaging or positron emission tomography (PET) imaging with [F-18] fluoromisonidazole (FMISO) [5,11,12]. Such measurements showed that, in addition to chronic hypoxia, tumour cells experience acute, intermittent and cycling episodes of hypoxia, even in close proximity to arterial vasculature [11,13]. There is emerging evidence that the different subtypes of hypoxia result in varied clinical outcomes, with acute or cycling hypoxia reported to be more aggressive when compared with chronic hypoxia [11]. Hence, the characterisation of neuroblastoma oxygenation is crucial for our understanding of its biological and clinical implications. However, despite advances in non-invasive techniques that monitor oxygen, clinical data on neuroblastoma are still missing.

The cellular response to hypoxia involves alteration of gene expression via the stabilisation and activation of the hypoxia inducible factor (HIF) [14]. This promotes cell survival by ensuring increased oxygen delivery and reduced oxygen consumption (increased glycolysis) [15–19]. Such metabolic change in tumours, classified as ‘deregulation of cellular energetics’, is an emerging hallmark of cancer [20]. Technological advances and low per-sample costs have contributed to the increase in metabolomic studies to elucidate the global metabolic changes occurring during cancer progression thereby defining novel oncological biomarkers [21]. Distinct metabolic profiles have already been described for certain tumours (e.g. [22]), and some metabolic biomarkers are already used in the clinic. Moreover, a number of drugs targeting metabolic pathways are employed as cancer treatments. However, routine elucidation of cancer metabolites as a general therapeutic/stratification strategy remains challenging, and the identification of specific and defined metabolic markers using preclinical and clinical approaches are required. A recent meta-analysis of clinical metabolomic studies encompassing 18 types of cancer confirmed the consistency of increased glycolysis and highlighted metabolites previously less associated with cancer such as histidine, tryptophan and 3-hydroxybutyric acid [23]. Although tumour tissue is more challenging to obtain and to prepare than common biofluid samples such as serum and urine, interest in direct tumour metabolomics has been increasing [24]. Specifically, the metabolome of neuroblastoma biopsy samples has recently been analysed to associate metabolites with patient age, prognosis, active disease and remission [25]*.*

We here aimed to elucidate, at a metabolic level, how hypoxic preconditioning alters the neuroblastoma cell metastatic phenotype, in order to identify potential new metabolic biomarkers of metastasis. We performed live oxygen measurements with a fibre-optic oxygen sensor to establish the oxygen levels in tumours formed by SK-N-AS neuroblastoma cells preconditioned in normoxia or hypoxia and implanted on to the CAM of chick embryos. We demonstrated that neuroblastoma oxygen levels are heterogeneous and fluctuate spatially and temporally with the exception of necrotic or haemorrhagic areas. Hypoxic preconditioning resulted in a lower overall mean oxygen level in the tumour tissue, however, more drastic changes were observed on the tumour metabolic profile, measured by 1D ^1^H NMR spectroscopy. Our analysis here demonstrates that the metabolic fingerprint of cell culture is only marginally comparable with the metabolic fingerprint of tumour tissues *in vivo*. Tumour samples have very high levels of choline/phosphocholine, taurine, glucose, fructose and lactate compared with cells, consistent with a higher level of glycolytic flux *in vivo* [26–31]. Hypoxic preconditioned tumours, which have a metastatic phenotype were associated with high levels of alanine, lactate, 3-hydroxybutyrate and decreased taurine levels, all in-line with glycolysis and cancer progression [32].

## Methods

### Cell culture

SK-N-AS cells (ECACC number: 94092302; authenticated in April 2018; passage number typically between 13 and 33) were cultured in Minimum Essential Medium supplemented with 10% (v/v) FBS and 1% (v/v) non-essential amino acids (all Thermo Fisher Scientific). Cells were maintained in a humidified incubator at 37°C, 5% CO_2_ and routinely tested for *Mycoplasma* infection. For preconditioning, cells were incubated for 3 days in either 21% O_2_ (normoxia) or 1% O_2_ (hypoxia) in a hypoxic workstation (Don Whitley Hypoxic Workstation, England). We ensured that the cells grew at a similar rate in both oxygen conditions and that they also had a comparable viability (Supplementary Figure S1A,B). GFP-labelled cells were used to implant on to the CAM for tumour development monitoring and precise tumour dissection. Non-labelled cells were used for generating the *in vitro* samples. We established that the metabolic fingerprint of labelled and non-labelled cells was comparable (not shown).

### CAM implantation

Fluorescently labelled NB cells preconditioned in 1 or 21% O_2_ for 3 days were implanted on the CAM of chick embryos at embryonic day 7 (E7) as described previously [33]. At E14, intratumoural oxygen tension was measured as described below.

### Oxygen measurements

Oxygen tension was monitored live *in vivo* in 80 tumour samples for a period up to 57 min and levels captured in 2 s intervals. Thirty-eight tumours originated from SK-N-AS cells precultured in normoxia, and 42 tumours from cells preconditioned in hypoxia. The measurements were carried out with a Microx 4 Fibre Optic Oxygen Meter fitted with a needle-type oxygen microsensor (PreSens, Germany). Of the 80 tumours, 57 measurements were performed with the probe attached to a micromanipulator (Presens, Germany) to record fluctuation with maximal stability.

### NMR samples

#### Cell harvesting

 SK-N-AS cells (1 × 10^6^) were incubated in either 21 or 1% O_2_ for 3 days in T25 flasks (Corning, U.K.). Cells were harvested with a 2 ml PBS wash, trypsinisation and suspension in 5 ml medium. A cell count was performed at harvesting time, and approximately 5–6 × 10^6^ cells were used for each condition. Supplementary Figure S1C shows a typical confluency at harvesting time for both normoxic and hypoxic conditions. In order to remove the residual culture medium, cells were gently washed in PBS by repeated cycles of centrifugation and supernatant discard. Upon centrifugation (1200×***g*** for 5 min), the supernatant was discarded and the pellet of cells was resuspended in 1 ml PBS, centrifuged (1200×***g*** for 5 min) and the pellet of cells was frozen immediately in liquid nitrogen and stored at −80°C until acetonitrile:water extraction.

#### Tumour harvesting

On E14, the tumours were dissected from the CAM followed by a PBS wash, stored in an Eppendorf tube and immediately frozen in liquid nitrogen. Samples were stored in −80°C until acetonitrile:water extraction.

#### Acetonitrile:water extraction

An ice-cold solution of 50% HPLC-grade acetonitrile and 50% double distilled water was added to the samples without any further washing: 400 µl for cells and 1.2 ml to tumours. Tumours were subsequently divided into technical triplicates following sonication. Samples were sonicated (MSE U.K. Soniprep 150 Plus Ultrasonic Disintegrator) for 3 × 30 s cycles at 10 kHz using a microtip probe. The lysed samples were vortexed (30 s) followed by centrifugation (5 min at 21500×***g***/4°C) to remove cell debris and the supernatants snap-frozen in liquid nitrogen for lyophilisation (Thermo Scientific) overnight. Due to the greater heterogeneity in tumour consistency, in order to clarify the supernatant from the fatty components of the tumour cell debris, the tumour samples were centrifuged and supernatant transferred to new Eppendorf tubes twice prior to freezing in liquid nitrogen and lyophilisation. Samples were stored in −80°C.

#### Sample preparation

 Two hundred microlitres of a solution comprising: 89.8% ^2^H_2_O, 10% (v/v) 1 mM sodium phosphate (pH 7.4), 0.1% (v/v) 100 mM selectively deuterated (d4) trimethyl silyl propionate (TSP) and 0.1% (v/v) 1.2 mM sodium azide, were added per sample. Samples were vortexed for 1 min followed by 2 min of centrifugation (20°C, 12000×***g***). The supernatant was pipetted into 3 mm NMR tubes and arranged in an NMR tube rack.

#### NMR acquisition

1D ^1^H NMR spectra were acquired on a Bruker Avance III HD 700MHz spectrometer equipped with a 5 mm TCI cryoprobe at pH 7.4 and 25°C. Two 1D ^1^H standard metabolomics experiments with optimal water suppression were acquired per sample; both are available in the Bruker pulse sequence library, the noesypr1d is based on the Nuclear Overhauser Effect (NOE) and the cpmgpr1d filters for small molecules via a Carr–Purcell–Meiboom–Gill (CPMG) sequence. The noesypr1d was acquired with 32 transients 20 ppm spectral width, 32K points, 10 ms mixing time and a 2.73 s acquisition time. The cpmgpr1d was acquired with 256 transients a 15 ppm spectral width, 32K points, 9.6 ms echo time and a 3.1 s acquisition time. Both spectra were acquired with a 4 s interscan delay, the protein-free baseline of the cpmgpr1d resulted in these spectra used for downstream analysis.

#### Spectral processing

Automated data processing, Fourier transformation and phasing were carried out in Topspin v3.2 software using standard Bruker routines. Chenomx v8.2 software was used for initial metabolite identification with manual confirmation to in-house standards for metabolite peaks found to be significantly variable. The spectra were then normalised to TSP reference signal and bucketed per peak into a matrix of metabolite peak intensities using AMIX v3.9.14. Statistical analysis was carried out using R (r-project.org) both standalone and via the metabolomics server MetaboAnalyst v3.0 [34].

### Statistical analysis

The prepared bucket table was subjected to univariate and multivariate analysis. Student’s *t* test was applied on the spectra after determining the data were parametric using ggplot function in R. Fold changes were calculated for median metabolite levels for buckets identified as significantly different by Student’s *t* test after *P*-value adjustment (Benjamini–Hochberg) to compensate for multiple testing. Unsupervised multivariate analysis of the spectra was carried out after using Principal Component (PC) Analysis (PCA). The data were scaled by mean centring of each variable followed by division of the square root of the standard deviation (SD) (Pareto scaling) before analysis. The PCA reduced the 300 spectral buckets of each sample to 15 PCs in the combined analysis of tumours and cells (explaining 95% of the variance). The loadings of each PCA were analysed to identify the most influential buckets (and thus metabolites) responsible for the clustering observed. Following unsupervised analysis of the four conditions, cells and tumours were analysed separately by Discriminant Analysis of PCs (DAPC) and Partial Least Squares-Discriminant Analysis (PLS-DA). Both PLS-DA and DAPC models were fit using the optimal number of components as determined by cross-validation using R packages ggplot and ade4. In order to elucidate the key metabolites in the separation of hypoxic and normoxic tumours or cells, a comparison of the most influential buckets for discriminant analysis was made both in the presence and absence of buckets attributed to unidentified metabolites.

## Results

### Intratumoural oxygen levels are heterogeneous, fluctuating and only marginally altered by a cellular hypoxic preconditioning

**Figure 1:**
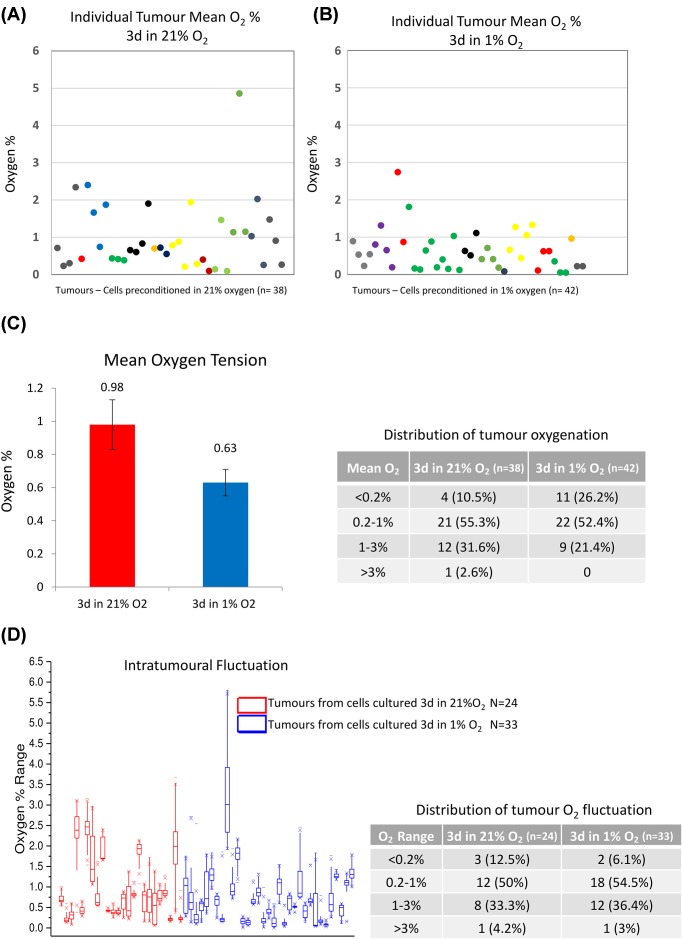
Mean oxygen tension measurements Intratumoural O_2_ tension readings were measured up to 57 min, using a fibre-optic oxygen measurement probe. The O_2_ levels plotted are the mean levels measured during the recording. (**A**) Mean oxygen tension of 38 tumours from normoxic cells, colour coded by experiment to allow sample matching with (**B**). (B) Mean oxygen tension of 42 hypoxic tumours. Measurements were performed over 14 batches of eggs. In two replicates, measurements were only obtained from one condition and could not be matched (light blue and purple). (**C**) The bar graph displays the overall mean oxygen tension ± S.E.M. of the normoxic and hypoxic tumours calculated as a mean of mean measurements in each sample type. *P*<0.05. The table shows the distribution of tumours in indicated oxygenation categories. Note that a few tumours had very low O_2_ levels, with no fluctuations and were taken in tumours which showed to have large blood pools or necrotic areas upon sectioning. (**D**) Oxygenation fluctuation was measured on a subset of tumours, using a micromanipulator to avoid measurement errors over time. The box plots display the range of oxygen tension in tumours during the measurement period (from 11 to 57 min). Each plot represents a single tumour. The table displays the percent of tumours in different fluctuation ranges (from non-fluctuating: <0.2% O_2_ difference over time, to highly fluctuating: >3% O_2_ difference over time) for tumours originating from normoxic or hypoxic preconditioned SK-N-AS cells.

Since we observed that SK-N-AS cell preconditioning in hypoxia for 3 days triggers a metastatic phenotype in tumours [8], we initially aimed to evaluate if the preconditioning could impact the actual tumour oxygenation. We performed precise live oxygen tension readings, with a fibre-optic sensor, in tumours formed on the CAM, from SK-N-AS cells precultured in either atmospheric (21% O_2_) or hypoxic (1% O_2_) environment for 3 days. Measurements were done on >35 tumours in each condition over 14 batches of eggs (see colour-coded experiments in Figure 1A,B) with readings being recorded at 2 s intervals for up to 57 min. Figure 1A,B shows a strong tumour to tumour variability, even within one experimental batch. The variation in tumour oxygenation was not correlated with tumour volume (Supplementary Figure S2A). Nevertheless, the majority of all tumours, regardless of the preconditioning conditions, yielded a mean O_2_ tension less than 1% O_2_ (72.5%; *n*=58) and a quarter (26.3%; *n*=21) were in the range of 1–3% mean O_2_ tension (Figure 1C, table). The mean O_2_ tension was marginally higher in tumours originating from cells precultured in normoxia compared with cells in hypoxia (mean O_2_ tension: 0.98% ± 0.15 S.E.M. compared with 0.63% ± 0.08 S.E.M., with statistical significance (*P*=0.04; Figure 1C). Despite the significant *P*-value, preconditioning the cells in a hypoxic environment did not have a major effect on the global tumour oxygenation (0.35% O_2_ difference), with tumours being globally hypoxic, similar to what was previously reported with oxygen measurements in various tumour types [35]. It has to be noted that several tumours had extremely low O_2_ levels, which were unusually very stable over time (see fluctuation box plot in Figure 1D). These tumours have not been excluded from the statistics here, but likely come from measurements in blood pools or necrotic areas, which we have subsequently observed upon dissection and sectioning.

The range of O_2_ level fluctuation over time was measured with nine sets of measurements that were performed for more than 10 min (between 11 and 57 min) using a micromanipulator to increase measurement stability (Figure 1D). Fluctuations of oxygen tension were found to be similar in both conditions with 62.5 and 60.6% of tumours fluctuating less than 1% in tumours formed from normoxic and hypoxic precultured cells, respectively (Figure 1D, table). Cycle length and fluctuation range were found to be independent of the duration of the measurement (Supplementary Figure S2B) and of the hypoxic/normoxic preconditioning. Together, these data confirm that oxygen levels in neuroblastoma tumours are fluctuating moderately and that the metastatic phenotype, acquired by the hypoxic preconditioning results in a slightly lower overall mean of oxygen levels without influencing the occurrence of cycling hypoxia. The measurements were limited to a period of 57 min and it is possible that these tumours experience a slower cycling time, which could not be detected in this experimental set-up.

### Metabolomic analysis of cell and tumour samples

**Figure 2:**
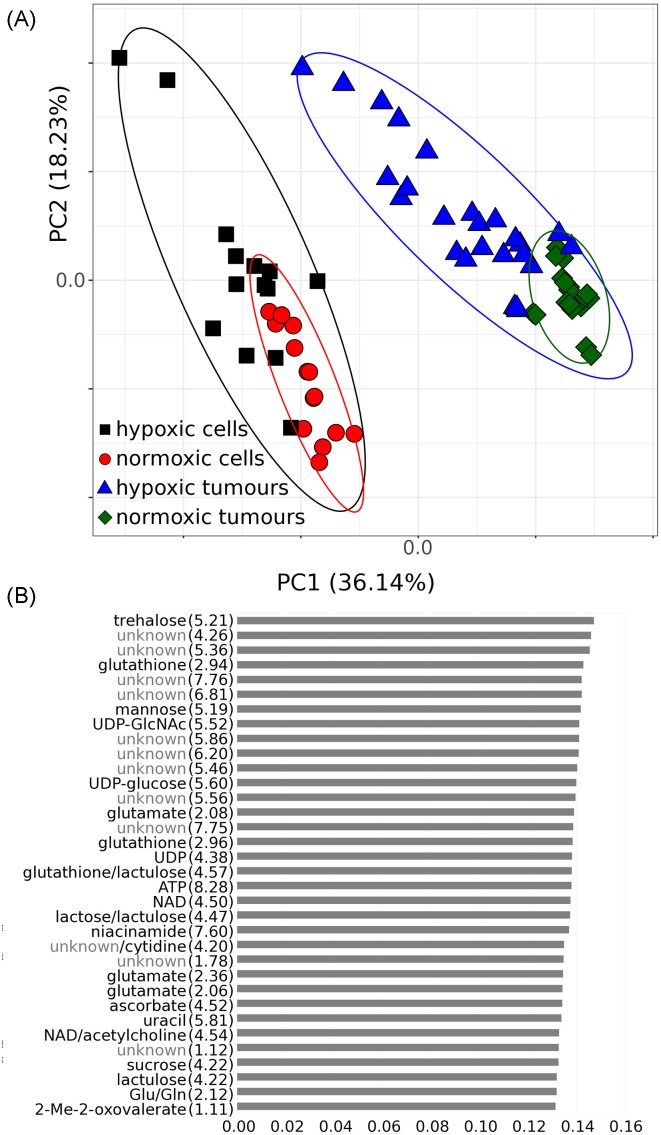
PCA of cell and tumour metabolite extracts (**A**) Scores plot shows greater variance in spectra of hypoxic samples (cells in black and tumours in blue) compared with samples cultured under normoxic conditions (cells in red and tumours in green). (**B**) Most influential buckets from PC1 and PC2 (combined). Numbers reported in parenthesis adjacent to variables represent the centre of the metabolite peak in ppm ± 0.005.

To further establish the intracellular modifications triggered by hypoxic preconditioning, we analysed the metabolic signature of neuroblastoma cells and neuroblastoma tumours formed on the CAM by normoxic or hypoxic preconditioned cells. NMR spectroscopy was used to identify the metabolic shift induced by hypoxia. All data are available on EBI (http://www.ebi.ac.uk/metabolights/MTBLS541). A quantile plot of all 1D ^1^H NMR spectra is shown in Supplementary Figure S3. The spectra were divided in 354 peaks or multiplets, of which 263 were assigned to a total of 98 metabolites. Data transformation using PCA exhibited variance among the four groups of spectra with cells or tumours cultured under hypoxic conditions more varied than those under normoxic conditions (Figure 2A). As expected, the metabolic profile of cells cultured *in vitro*, was different and well separated from cells forming tumours on the CAM *in vivo*. Analysis of the loadings of metabolites that are most influential in the first and second PCs indicate that trehalose, glutathione and mannose together with several unidentified metabolites contribute greatest to the variance between spectra (Figure 2B). The chemical shifts of the unknown components are consistent with saccharides (whose anomeric 1H shifts are observed between 4 and 6 ppm) and/or molecules containing aromatic rings (shifts observed between 6 and 10 ppm).

**Figure 3:**
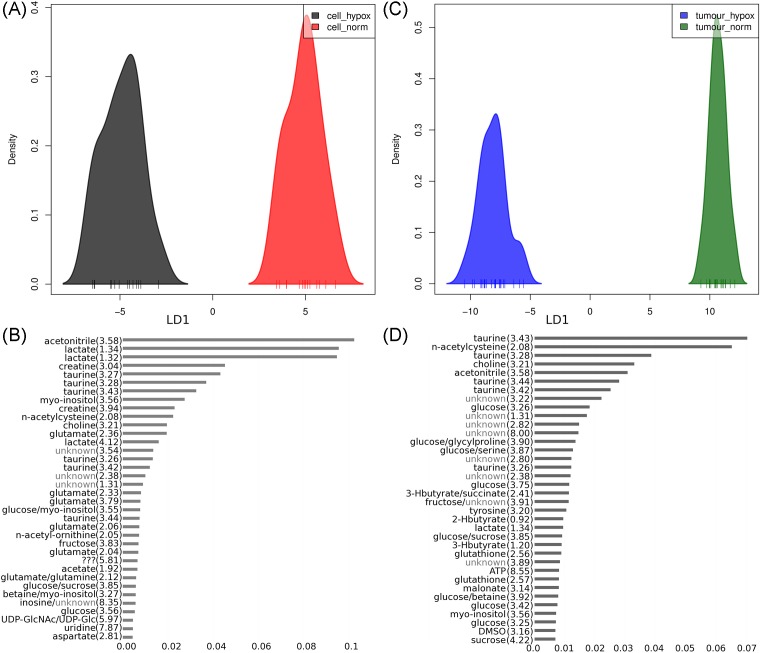
DAPC plots of total NMR metabolite profiles. Plots show profiles from cells (**A**,**B**) and tumours (**C**,**D**) under hypoxic and normoxic conditions. Linear discriminant one for (A) hypoxic and normoxic cells shown in black and red respectively, and (C) hypoxic and normoxic tumours shown in blue and green respectively. Most influential buckets for the DAPC models for cells (B) and tumours (D). Numbers reported in parenthesis adjacent to variables represent the centre of the metabolite peak in ppm ± 0.005.

 DAPC was carried out to identify peaks and therefore metabolites responsible for spectral changes between the hypoxic and normoxic cell and tumour models (Figure 3). DAPC analysis required just one linear discriminant in order to distinguish between hypoxia and normoxia in either cells (Figure 3A) or tumours (Figure 3C). A comparison of the most influential metabolite signals (also referred to as buckets) responsible for the separation leads to the identification of metabolites that are at different abundances between normoxic and hypoxic conditions either in one or in both cells, and tumour systems. Taurine, choline, lactate, *myo*-inositol, n-acetylcysteine and acetonitrile appear in both models. For example, the relative intensities for representative metabolite peaks for taurine are 17.89 ± 4.3 in normoxic cells compared with 21.61 ± 2.24 in hypoxic cells, and 35.45 ± 9.49 in normoxic tumours compared with 23.07 ± 5.27 in hypoxic tumours. Creatine, glutamate, uridine, n-acetyl-ornithine and aspartate appear in the 10% most influential buckets of the cellular DAPC model only (Figure 3B). Whereas tyrosine, 2-hydroxybutyrate, 3-hydroxybutyrate, ATP, malonate and glutathione appear in the most influential metabolite signals of the tumour DAPC model only (Figure 3D). Interestingly, DAPC analysis of the four conditions in one model exhibited greater differences between hypoxic and normoxic tumour profiles than between hypoxic and normoxic cell profiles (Supplementary Figure S4). With the two-condition model presented in Figure 3, we identified taurine, choline, lactate, *myo*-inositol, n-acetylcysteine and acetonitrile as being influential in normoxia-hypoxia group separation.

### Discriminant analysis of specific metabolites to distinguish hypoxia from normoxia

**Figure 4:**
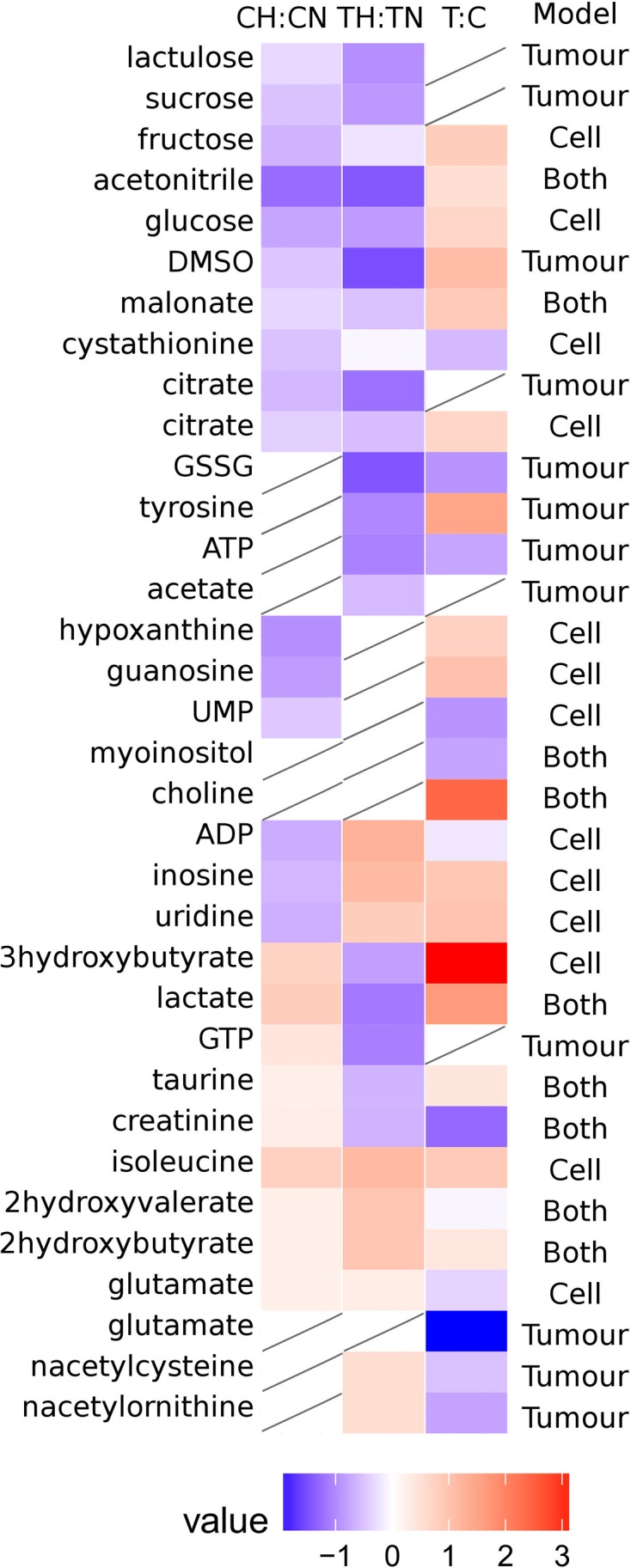
Fold-change heatmap for 33 shortlisted metabolite peaks that are used to distinguish normoxic from hypoxic conditions in either cells or tumours Heatmap colour only shown where metabolite levels are deemed significantly different below a *P*-value 0.05 (*P*-values adjusted for multiple testing by Benjamini–Hochberg method).

In order to elucidate the key metabolites in the separation of hypoxic and normoxic tumours or cells, a comparison of the most influential buckets for discriminant analysis was made. From the DAPC loadings, 22 metabolites were identified for cells and 21 metabolites identified for tumours with only 9 of the metabolites common to both systems. This shortlist of 34 metabolite buckets is shown on the heatmap (Figure 4), with many exhibiting *P*-values (adjusted for false discovery rate) consistent with significantly different metabolite levels between cells or tumour conditions.

**Figure 5:**
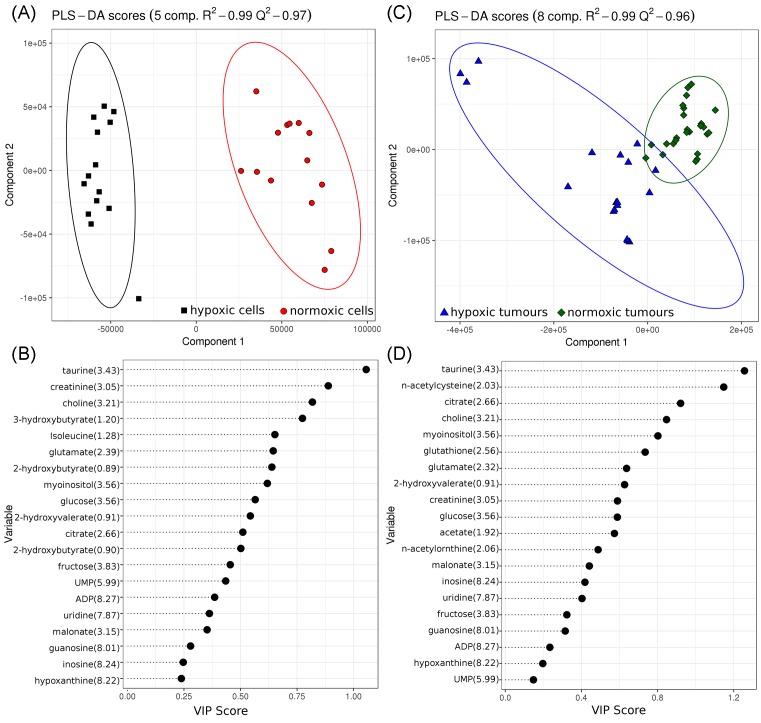
PLS-DA models. Models use shortlist of metabolites for either cells (**A**,**B**) or tumours (**C**,**D**) under hypoxic (red or green) and normoxic (black or blue) conditions. Variable influencing permutation (VIP) scores (B,D) rank the influence of the metabolites over the model (for all components).

The shortlisted metabolites were then used exclusively to perform a PLS-DA between hypoxic and normoxic conditions for either cells or tumours. The PLS-DA models for both cells and tumours were optimally fit with five components (cells) and eight components (tumours) and yielded well-defined separation between hypoxic and normoxic conditions with *R^2^* and *Q^2^* of 0.99 and 0.97 respectively, for the cellular model and 0.99 and 0.96 respectively, for the tumour model (Figure 5). Greater variation in the metabolite profile of tumour samples (as observed in Figure 2) may account for the slightly lower predictive *Q^2^* score.

### Contrasting metabolite fingerprints between neuroblastoma cells and tumours formed on the CAM

**Figure 6:**
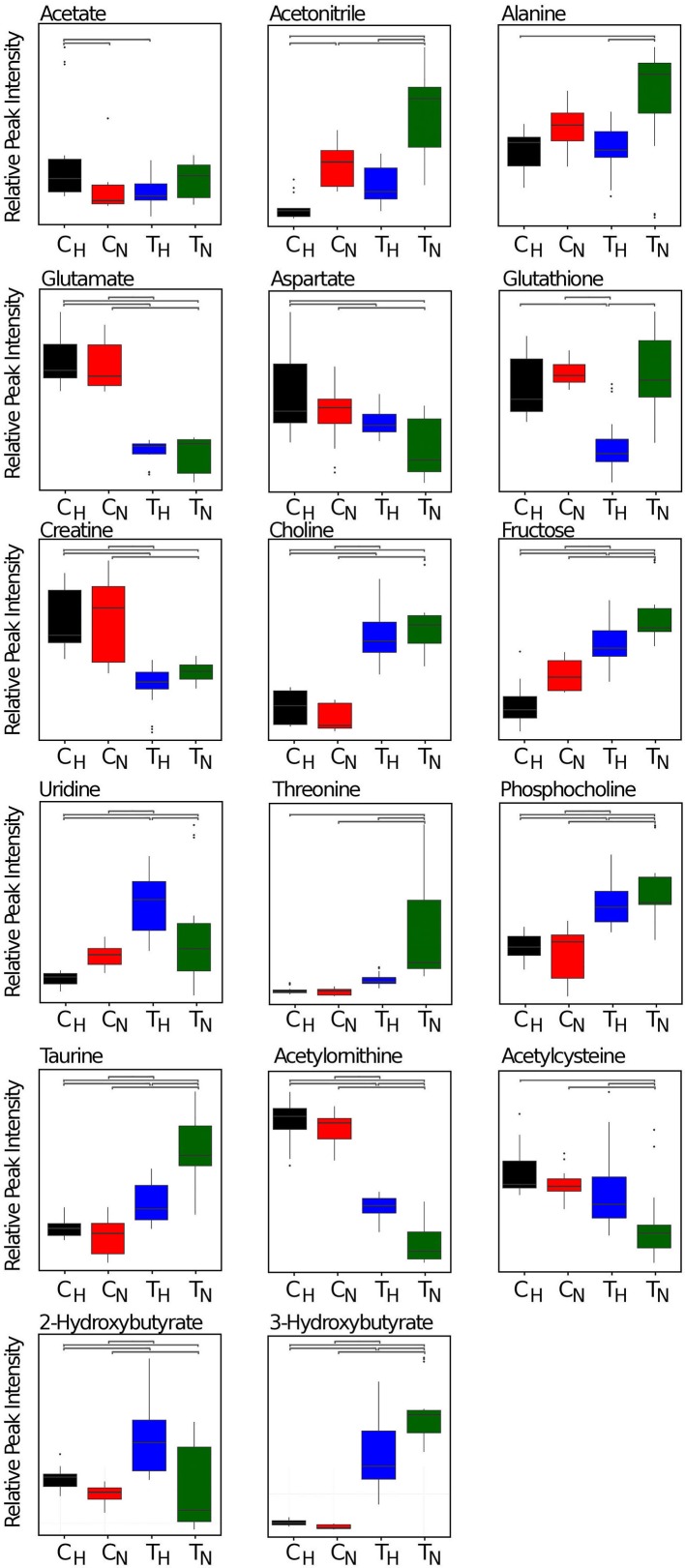
Box plots of representative metabolite peaks listed in Figure 4 for normoxic SK-N-AS cells (C_N_), hypoxic SK-N-AS cells (C_H_), tumours from normoxic cells (T_N_) and tumours from hypoxic cells (T_H_) Bars represent significant difference between groups (Tukey’s post-hoc analysis, adjusted *P*-value <0.05). The relative intensity and S.D. for representative metabolite peaks are also provided as numbers in Supplementary Table S1.

Most metabolite levels between hypoxic and normoxic conditions for the discriminating molecules exhibited differing trends between the cellular and tumour conditions (Figures 4 and 6). Of the 34 metabolite buckets inspected, the majority (*n*=10) exhibited lower levels under hypoxic conditions including lactulose, sucrose, fructose, acetonitrile, glucose, DMSO, malonate, cystathione and citrate. It is likely that DMSO and acetonitrile are products of the culture media and extraction lysate respectively. Multiple carbohydrates, citrate and ATP were also lower under hypoxic conditions, consistent with highly productive glycolysis and lower mitochondrial activity (citric acid cycle) in hypoxic conditions [36].

Five metabolites (3-hydroxybutyrate, lactate, GTP, taurine and creatinine) were elevated in normoxic tumours compared with hypoxic tumours whereas, conversely, were elevated in hypoxic cells when compared with normoxic cells. In both cell and tumour extracts, four metabolites (isoleucine, 2-hydroxyvalerate, 2-hydroxybutyrate and glutamate) were at significantly higher levels under hypoxic conditions (Figure 6 and Supplementary Table S1). Unsurprisingly, the majority of metabolites are at higher levels in tumours than cells irrespective of oxygen levels with notable exceptions being medium components glutamate, creatinine and ATP. *Myo*-inositol and choline were not significantly altered in either tumour or cell system, however multivariate analysis has identified them as discriminant between hypoxic and normoxic conditions. This was evinced by the high variable influencing permutation (VIP) attributed to each in PLS-DA models, highlighting the importance of subtle distinctions not identified by crude univariate analysis.

Altogether, there are many differences between the cellular and tumour samples, with changes triggered by the hypoxic incubation occurring in opposite ways. The most notable one being the glycolytic pathway triggered, as expected, by the hypoxic incubation in cell culture (high levels of lactate), but not in tumours. However, this could be explained by the fact that the tumours show a much greater increase in the glycolytic pathway compared with the hypoxic cells (lactate and glucose). Nevertheless, despite many discrepancies between the *in vitro* and *in vivo* samples, some metabolite changes are consistent between the two, especially the lower levels of carbohydrates (fructose, sucrose, lactulose) in hypoxic cells and tumours in agreement with a productive glycolysis.

## Discussion

Neuroblastoma tumour formation on the CAM and its tuneable metastasis by hypoxic preconditioning, makes the chick embryo an ideal model to study the molecular mechanisms of hypoxia-induced metastasis. We have previously demonstrated that a 3-day hypoxic preconditioning of cells, prior to tumour formation elicits a clear metastatic phenotype, by triggering tumour cells intravasation, and by lowering their migration speed in blood vessels to facilitate extravasation [8]. We here show that these phenotypes are accompanied by some significant changes in the tumour cell metabolome, including high levels of ketones (3-hydroxybutyrate), lactate, and phosphocholine. Since the preconditioning does not strongly affect the global tumour oxygenation (0.35% O_2_ difference on average) or the O_2_ fluctuation over time (Figure 1), the metabolomic signature observed in the tumours originating from hypoxic cells is likely due to the preconditioning rather than the actual tumour oxygen levels.

### Tumour oxygenation: heterogeneity in space and time

Quantification of O_2_ levels in tumours has previously been reported to characterise the extent of tumour hypoxia in various malignancies, in both human patients and animal models [35,37–45]. However, no oxygen tension data in neuroblastoma tumours are available. Our oxygen measurements and observed fluctuations correspond to others previously reported for glioblastoma and other brain tumours in humans with median values reported to be 4.9 mmHg (0.69% O_2_), 5.6 mmHg (0.78%O_2_) and 22.24 mmHg (3.11%O_2_) [6,45,46]. We aimed to detect and quantify the cycling pattern and fluctuation in O_2_ levels, which were previously reported in mice models by Dewhirst et al. [3,47,48]. Although we did not identify clear cycles of hypoxia-reoxygenation (Supplementary Figure S5), we clearly observed variations over time within a 1% range in O_2_ levels in most of the cases (Figure 1D,E). These observations are in-line with others, which found that 8–20% of tumour cells experience oxygen fluctuation when measured with hypoxic markers [47]. Our higher number of fluctuating tumours is likely due to the fact that we performed direct O_2_ measurements, instead of relying on less sensitive hypoxic markers as in [36]. The variations observed are also comparable with the previously observed phenomenon of intermittent or cycling, hypoxia in human and animal tumour models [3,49,50].

### Metabolic signature of hypoxia in cells and tumours

Previous studies have compared the metabolomic fingerprint of cells cultured in normoxia and hypoxia at different time points [51,52]. In both cases, measurements were performed at both short (4 or 6 h) and long (48 h) hypoxic incubation. It is difficult, however, to find a strong consensus between the different cell lines. The increased level of lactate and lower levels of *myo*-inositol from Tsai et al. [52] is comparable with our observations. However, our results show that these changes detected in cells are different in the tumour samples. The several discrepancies that we observed between cells and *in vivo* tumours point to the necessity to carefully choose the cancer model and question the usefulness of the *in vitro* data. Comparisons between *in vitro* and *in vivo* metabolic profiles of cells subjected to hypoxia demonstrate the remarkable differences in metabolic alterations by the hypoxic preconditioning in the two different situations. One major difference, which might account for the varying results between cell samples and tumours, beyond the different microenvironment, is that with cell samples, only the intracellular metabolites were measured, with extracellular component washed away with the medium. In tumour samples, however, there will be a mix of tumour cells, stromal cells and although tissue is washed, it is likely to contain residual secreted metabolites.

The higher levels of taurine found in hypoxic cells compared with normoxic ones is in-line with observations in glioblastoma cell lines [51] and might be an adaptation mechanism for cells to survive better in hypoxic conditions. Indeed, it has been shown that taurine can protect human neuroblastoma cells in culture [53] and may exert a protective function against hypoxia/reoxygenation by reducing the endoplasmic reticulum stress [54]. In addition, taurine administered during hypoxia can reduce the cell damage due to O_2_ deficiency [55]. High taurine levels were also found in MYC-N amplified neuroblastoma cells lines in a study by Peet et al. [56], again pointing to a link between taurine levels and tumour aggressive phenotype. However, *in vivo*, taurine levels were decreased in tumours formed by hypoxic cells, which are the most metastatic ones, hence discrediting the *in vitro* findings. Nonetheless, this is consistent, with the epithelial-mesenchymal transition inhibitory effect of taurine in human prostate cancer cells [57], and suggest that the decreased levels, would be favourable to the metastatic process.

We found that the non-essential amino acid glutamate was consistently higher in hypoxic cells and in tumours from hypoxia-preconditioned cells. This is consistent with the role of glutamate, beyond being an excitatory neurotransmitter, in the regulation of proliferation, migration, and survival of normal neuronal progenitor cells [58,59] and neoplastic cells [60].

### The tumour metabolome

Some metabolite levels in the tumour samples correlated well with existing literature on tumour metabolomics. The low levels of creatine and *myo*-inositol and high levels of lactate and choline in the tumour samples are in-line with the level of expression of these metabolites in tumour tissues, shown by a recent meta-analysis of the metabolic profile of 18 tumour types [23]. However, the lower levels of acetate and creatine in hypoxic metastatic tumours is surprising as high levels of acetate and creatine were found in stage IV human neuroblastoma biopsy samples [25]. Interestingly, hypoxic cells seemed to share more elevated metabolite levels observed in human neuroblastoma tissue [25] than the CAM tumours. The end products of aerobic glycolysis: 3-hydroxybutyrate and lactate both have the same profile of regulation. They are at higher levels in hypoxic cells, consistent with the high glycolytic activity induced by the hypoxic environment, and they are observed at much higher levels in tumours compared with cells. Yet, they are not further elevated in the tumours formed by hypoxia-preconditioned cells compared with tumours from normoxic cells, potentially due to the very high levels already reached in tumours. Administration of 3-hydroxybutyrate increased tumour growth by approximately 2.5-fold in a model of MDA-MB-231 breast cancer xenografts [61]. In this model, both 3-hydroxybutyrate and l-lactate functioned as chemoattractants and stimulated the migration of epithelial cancer cells while lactate stimulated the formation of lung metastases. Therefore, the high levels of ketones and lactate found in our tumour model is consistent with their previously reported role in fuelling tumour growth and metastasis [61].

In conclusion, we have demonstrated that neuroblastoma tumours formed on the CAM of the chick embryo have a heterogeneous profile of oxygenation in time and space, but are mostly hypoxic (<1.5% O_2_) as previously reported in other human tumour types. We identified a very specific metabolite profile in cells and in tumours and could discriminate well the effects of hypoxic incubation *in vitro* or hypoxic preconditioning *in vivo*. While the most notable effect of hypoxia *in vitro* was the expected increased levels of the metabolites associated with glycolysis; *in vivo*, the effects ranged from a decrease in carbohydrates, ATP and taurine and an increase in ADP, isoleucine and glutamate, and their direct association with neuroblastoma metastasis will need to be further investigated.

## Supporting information

**supplementary Figure 1:** 

**supplementary Figure 2:** 

**supplementary Figure 3:** 

**supplementary Figure 4:** 

**supplementary Figure 5:** 

**Table 1:** Median relative intensity and standard deviation for representative metabolite peaks shortlisted for discriminant analysis

## Abbreviations

CAM: chorioallantoic membrane
DAPC: discriminant analysis of principal component
PC: principal component
PCA: PC analysis
PLS-DA: partial least squares-discriminant analysis
TSP: trimethyl silyl propionate
NB: neuroblastoma
NOE: Nuclear Overhauser Effect
CPMG: Carr–Purcell–Meiboom–Gill
SD: standard deviation
