# Supplementary material for: Effects of hypoxic preconditioning on neuroblastoma tumour oxygenation and metabolic signature in a chick embryo model
Source: Biosci Rep. 2018 Aug 29;38(4):BSR20180185. doi: 10.1042/BSR20180185 (PMC6131206; doi:10.1042/BSR20180185)
Supplement: Supplementary file 1 [file bsr20180185_Supp1.pdf]

Supplementary Figure 1

A

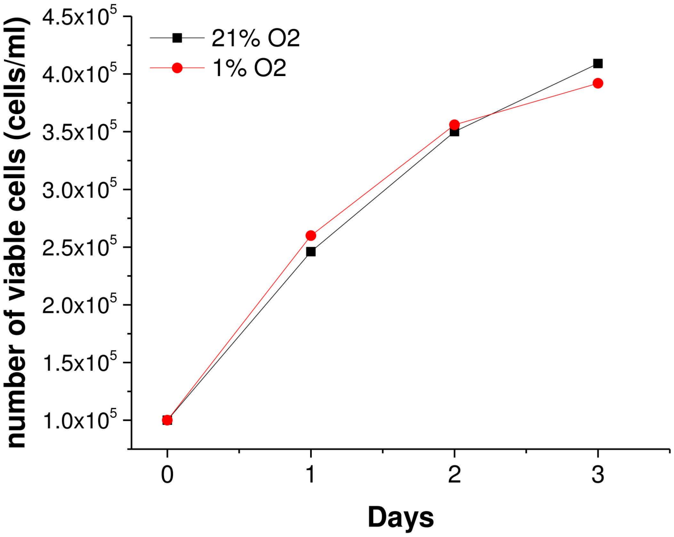

B

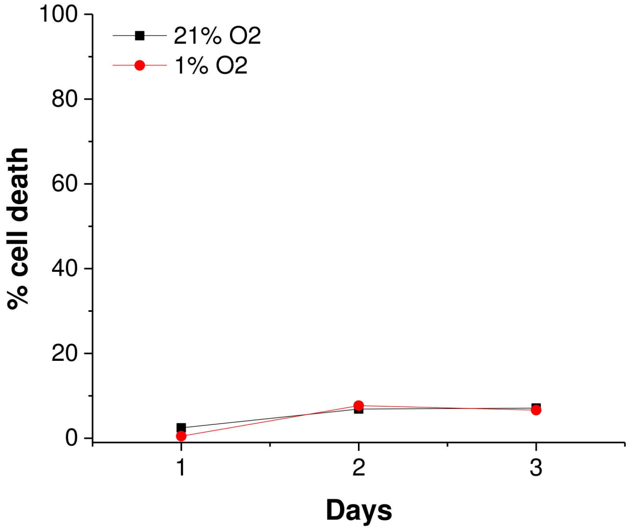

C

21% O_2 1% O_2

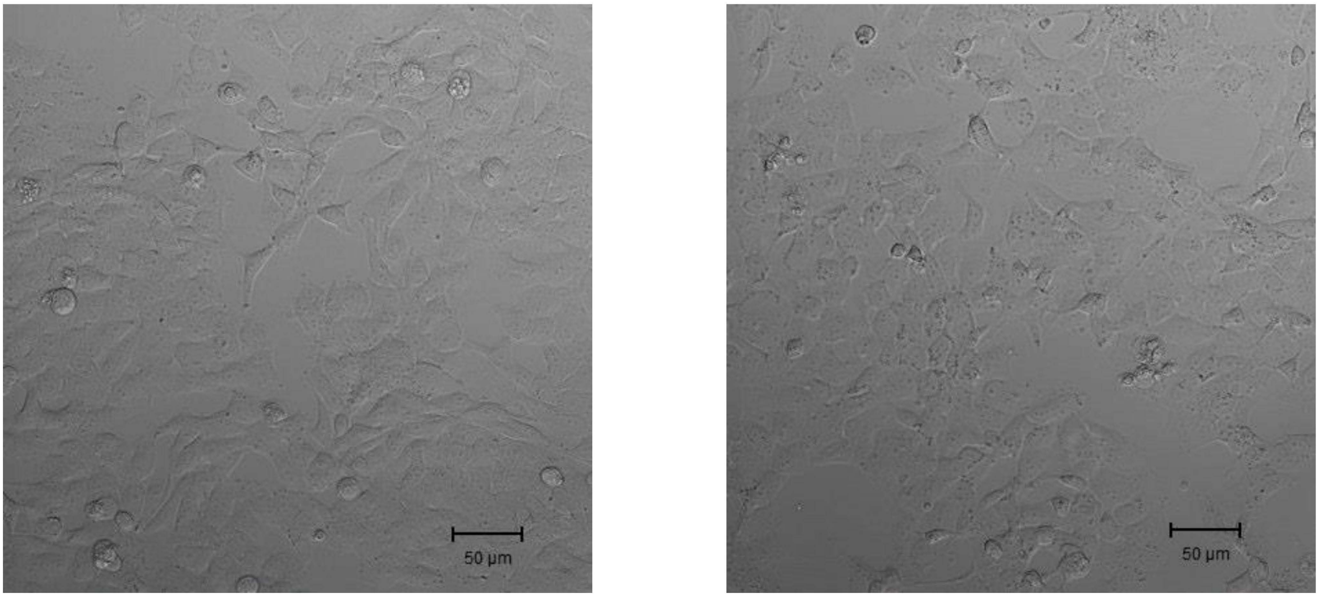

Supplementary Figure 2

A

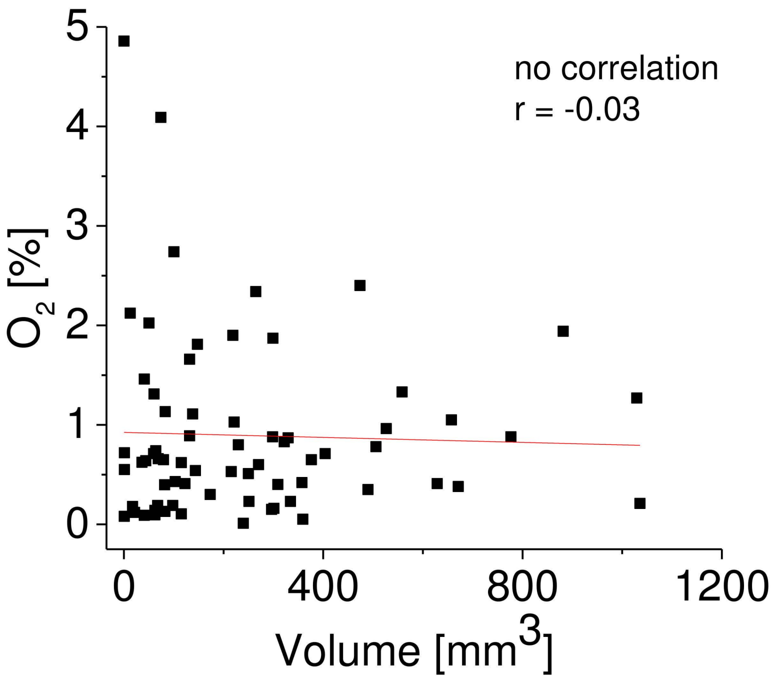

B

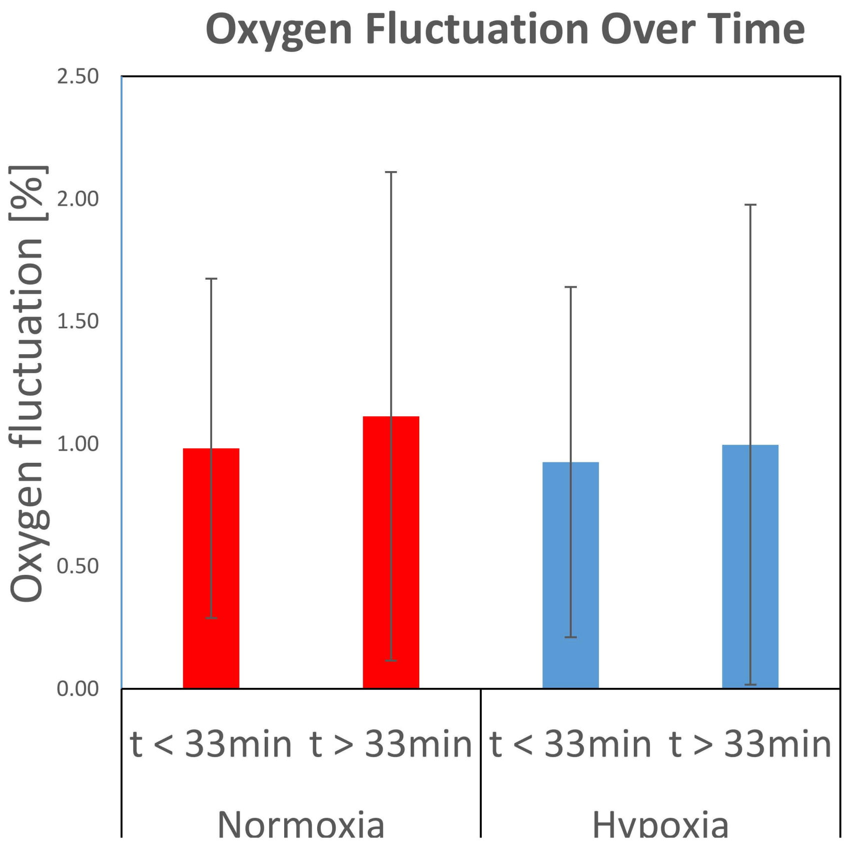

Supplementary Figure 3

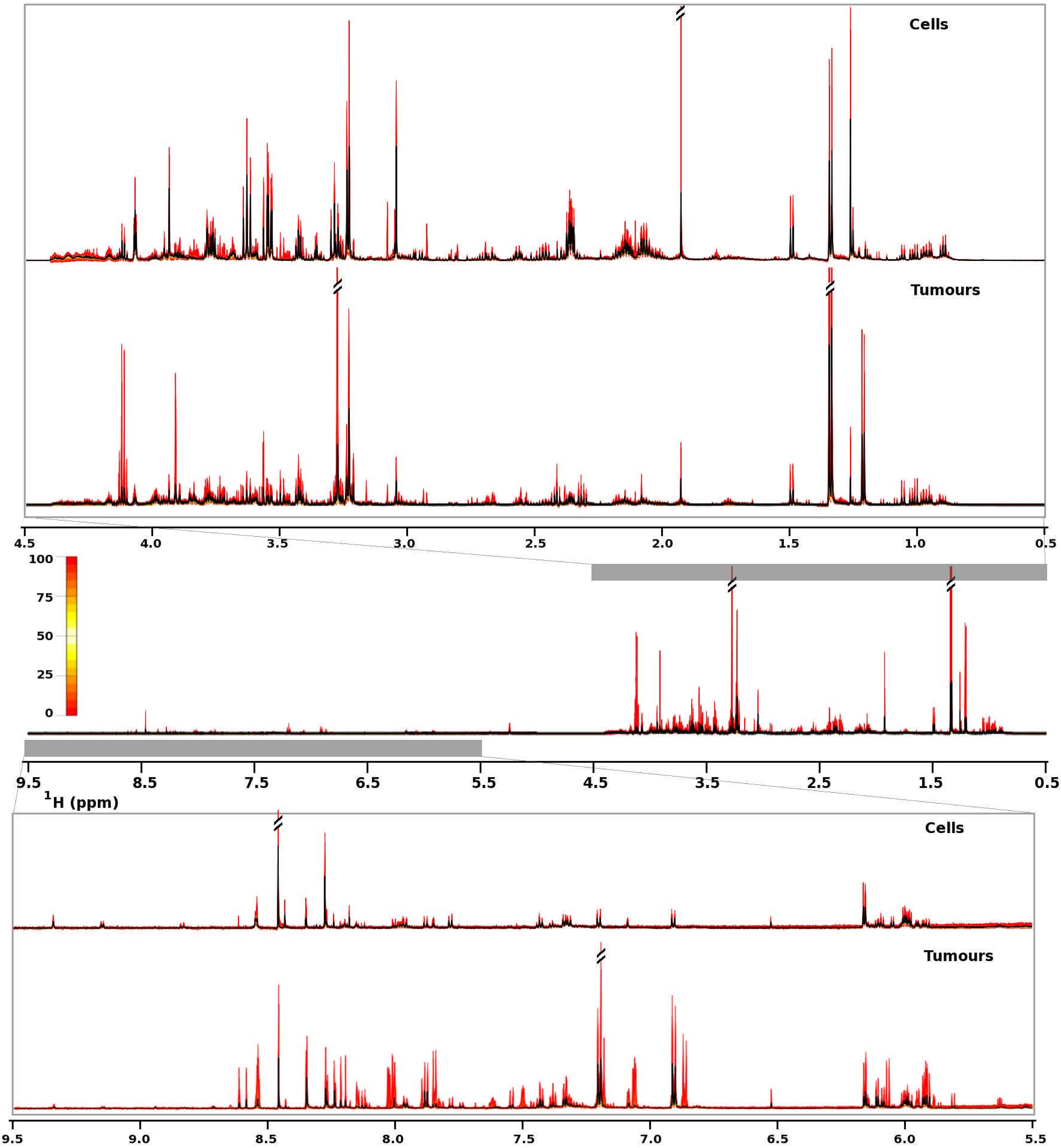

Supplementary Figure 4

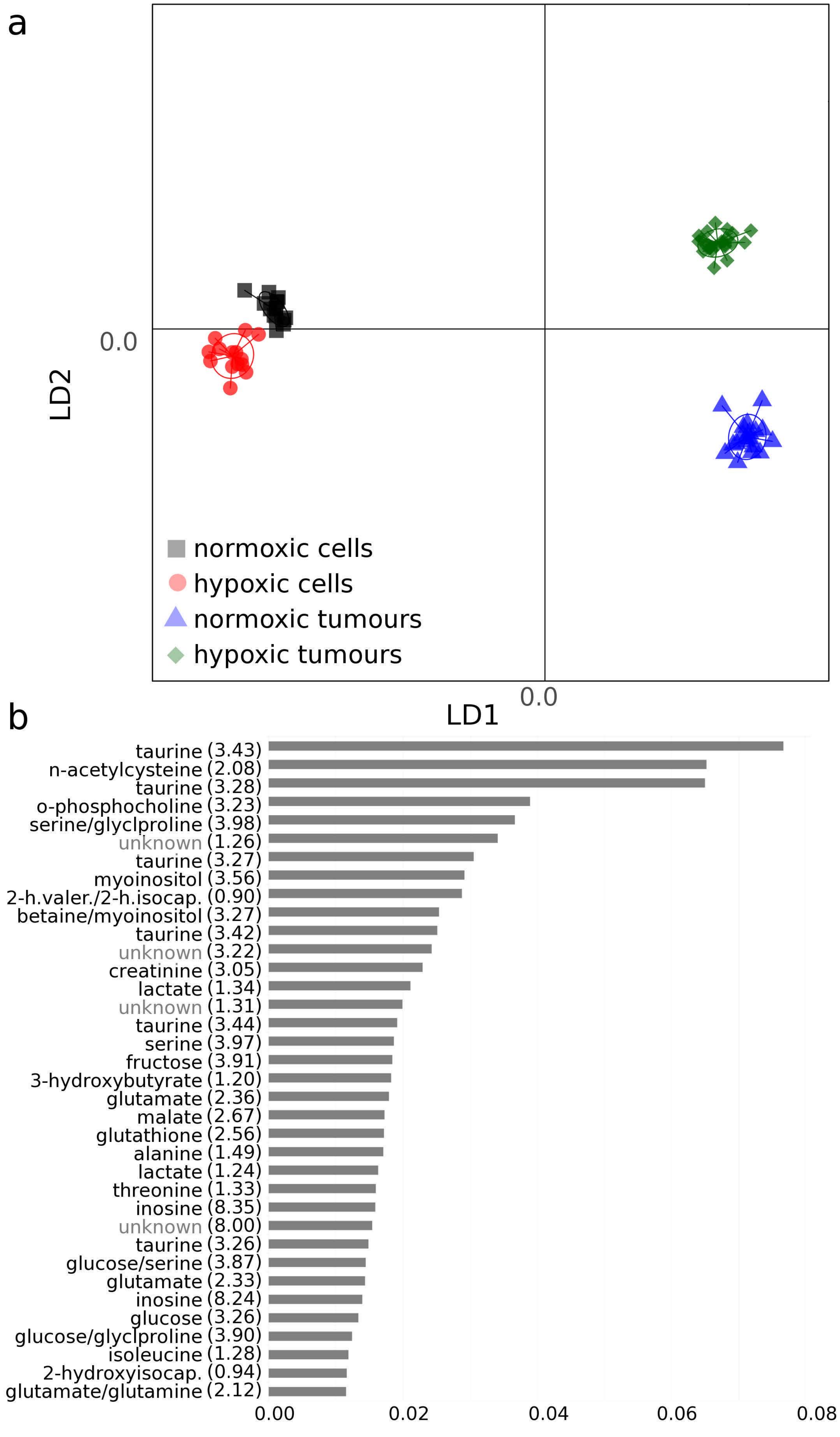

Supplementary Figure 5

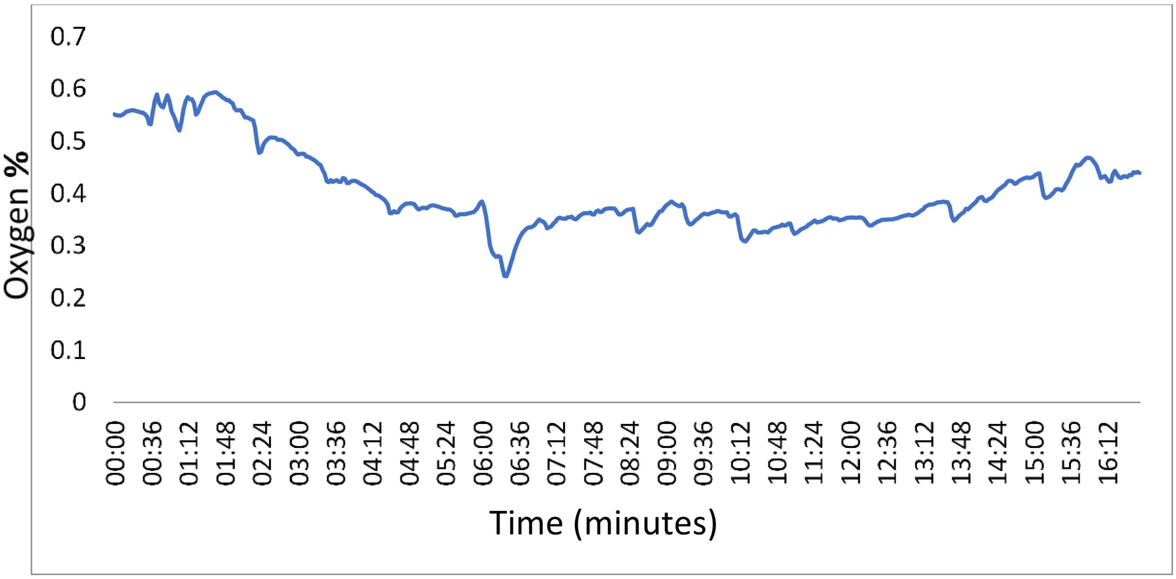

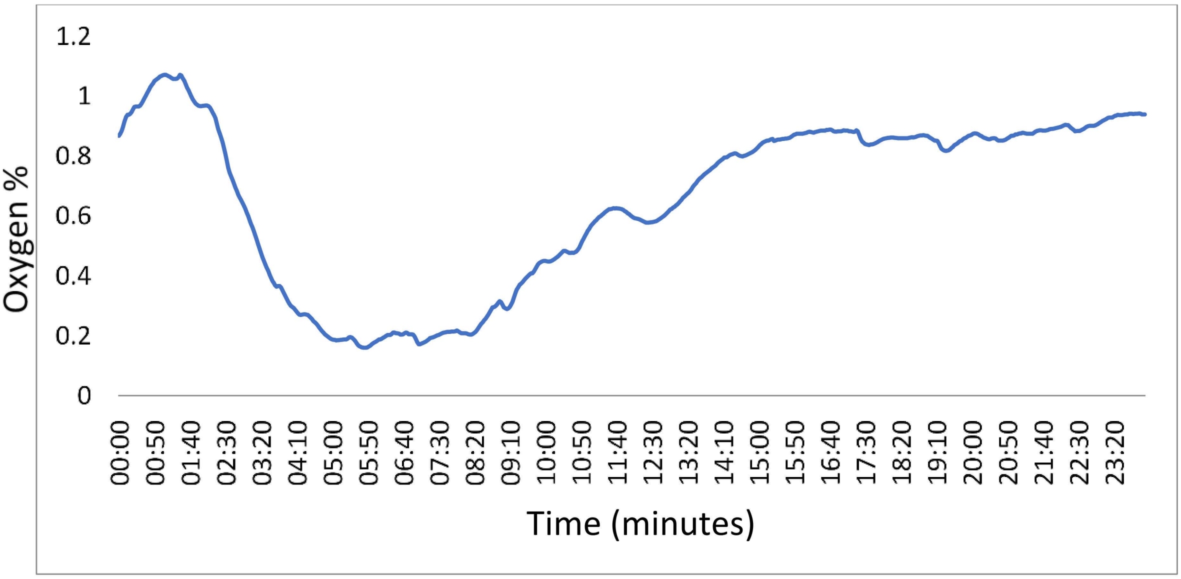

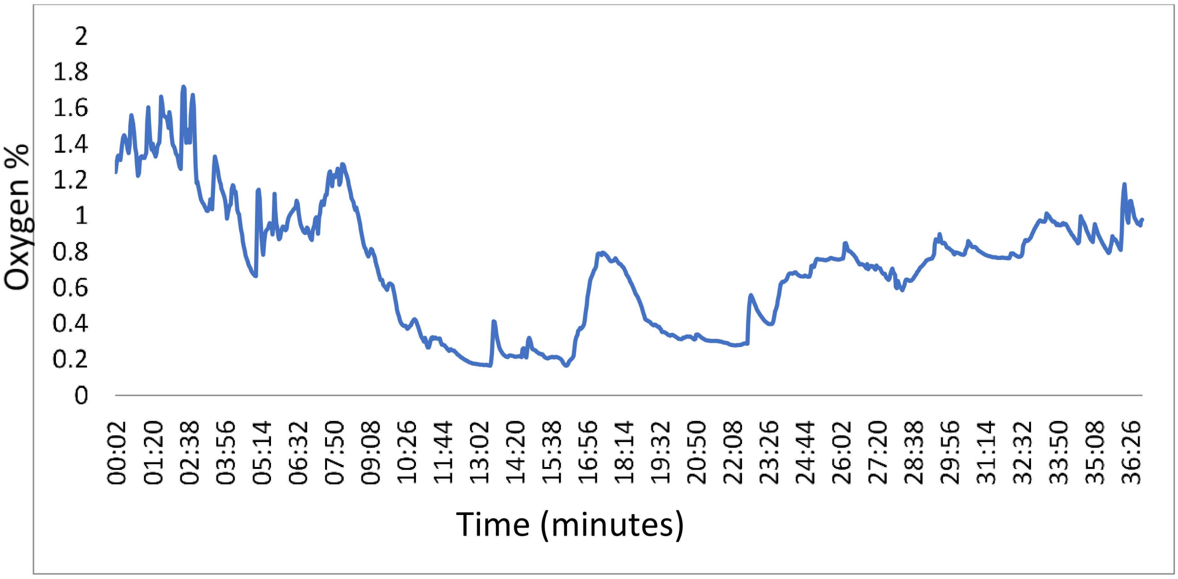

Table 1: Median relative intensity and standard deviation for representative metabolite peaks shortlisted for discriminant analysis

	CN	CH	TN	TH
**ATP**	2.19 (0.36)	2.09 (0.32)	2.42 (0.73)	0.89 (0.28)
**ADP**	1.87 (0.31)	1.16 (0.24)	0.57 (0.72)	1.39 (0.25)
**inosine**	1.17 (0.4)	0.77 (0.24)	0.95 (0.56)	2.12 (0.77)
**hypoxanthine**	0.61 (0.13)	0.32 (0.15)	0.85 (0.89)	0.83 (0.23)
**GTP**	0.97 (0.17)	1.31 (0.23)	0.97 (0.79)	0.46 (0.18)
**guanosine**	0.99 (0.2)	0.56 (0.16)	1.59 (1.56)	1.3 (0.99)
**uridine**	1.44 (0.26)	0.9 (0.17)	1.57 (1.24)	2.78 (0.73)
**tyrosine**	1.28 (0.21)	1.29 (0.38)	4.42 (1.1)	2.19 (0.87)
**UMP**	3.53 (0.31)	2.57 (0.52)	1.66 (0.28)	1.66 (0.32)
**lactulose**	3.84 (0.67)	3.07 (1.51)	5.13 (0.74)	2.66 (0.93)
**sucrose**	3.96 (1.13)	2.79 (1.48)	4.93 (0.76)	2.71 (0.9)
**fructose**	3.69 (0.63)	2.37 (0.89)	5.7 (1.12)	4.88 (0.92)
**acetonitrile.**	38 (9.72)	16.1 (5.54)	66.13 (18.89)	24.82 (7.79)
**glucose**	4.35 (1.13)	2.58 (1.03)	8.93 (2.36)	4.99 (1.46)
**myoinositol**	41.36 (8.67)	33.95 (11.3)	22.73 (5.28)	25.57 (5.17)
**taurine**	17.89 (4.3)	21.61 (2.24)	35.45 (9.46)	23.07 (5.27)
**choline**	4.28 (3.38)	9.46 (4.3)	31.02 (8.51)	26.75 (7.13)
**DMSO.**	1.6 (0.23)	1.16 (0.26)	5.41 (6.72)	1.92 (0.71)
**malonate**	3.1 (0.39)	2.46 (0.37)	6.66 (0.77)	4.67 (0.63)
**cystathionine**	2.02 (0.41)	1.41 (0.5)	1.17 (0.42)	1.11 (0.29)
**creatinine**	9.07 (2)	11.09 (2.95)	5.44 (0.88)	3.48 (0.55)
**citrate**	5.63 (1.33)	3.74 (1.28)	7.46 (3.02)	3.25 (1.13)
**glutathione**	8.82 (0.81)	7.39 (1.92)	8.51 (2.57)	4.07 (1.56)
**citrate**	5.87 (0.71)	4.49 (1.13)	12.36 (4.05)	8.39 (2.09)
**glutamate**	5.3 (0.6)	6.31 (0.64)	4.15 (0.51)	5.15 (0.88)
**nacetylcysteine**	26.23 (3.56)	26.71 (6.29)	14.97 (8.28)	21.93 (8.83)
**nacetylornithine**	8.62 (0.62)	8.88 (0.83)	3.69 (0.71)	5.46 (0.38)
**acetate**	16.31 (10.76)	26.59 (25.76)	27.8 (7.97)	18.61 (6.7)
**lactate**	41.23 (12.32)	73.65 (19.66)	309.88 (200.1)	141.61 (45.2)
**isoleucine**	2.24 (0.52)	3.82 (1.17)	3.72 (0.71)	8.31 (4.93)
**3-hydroxybutyrate**	3.62 (0.97)	6.07 (1.38)	57.7 (14.07)	33.05 (18.42)
**2-hydroxyvalerate**	4.21 (0.48)	4.98 (0.45)	3.25 (0.68)	6.22 (2.27)
**2-hydroxybutyrate**	5.3 (0.52)	6.31 (0.7)	4.95 (1.69)	9.55 (3.26)
